# Plasmodium falciparum Genetic Diversity in Coincident Human and Mosquito Hosts

**DOI:** 10.1128/mbio.02277-22

**Published:** 2022-09-08

**Authors:** Zena Lapp, Andrew A. Obala, Lucy Abel, David A. Rasmussen, Kelsey M. Sumner, Elizabeth Freedman, Steve M. Taylor, Wendy Prudhomme-O’Meara

**Affiliations:** a Duke Global Health Institute, Duke University, Durham, North Carolina, USA; b School of Medicine, College of Health Sciences, Moi University, Eldoret, Kenya; c Academic Model Providing Access to Healthcare (AMPATH), Eldoret, Kenya; d Department of Entomology and Plant Pathology, North Carolina State University, Raleigh, North Carolina, USA; e Bioinformatics Research Center, North Carolina State University, Raleigh, North Carolina, USA; f Department of Epidemiology, Gillings School of Global Public Health, University of North Carolina, Chapel Hill, North Carolina, USA; g Division of Infectious Diseases, School of Medicine, Duke University, Durham, North Carolina, USA; NIAID/NIH

**Keywords:** malaria, *Plasmodium falciparum*, transmission, comparative genomics, genetic diversity

## Abstract

Population genetic diversity of Plasmodium falciparum antigenic loci is high despite large bottlenecks in population size during the parasite life cycle. The prevalence of genetically distinct haplotypes at these loci, while well characterized in humans, has not been thoroughly compared between human and mosquito hosts. We assessed parasite haplotype prevalence, diversity, and evenness using human and mosquito P. falciparum infections collected from the same households during a 14-month longitudinal cohort study using amplicon deep sequencing of two antigenic gene fragments (*ama1* and *csp*). To a prior set of infected humans (*n* = 1,175/2,813; 86.2% sequencing success) and mosquito abdomens (*n* = 199/1,448; 95.5% sequencing success), we added sequences from infected mosquito heads (*n* = 134/1,448; 98.5% sequencing success). The overall and sample-level parasite populations were more diverse in mosquitoes than in humans. Additionally, haplotype prevalences were more even in the P. falciparum human population than in the mosquito population, consistent with balancing selection occurring at these loci in humans. In contrast, we observed that infections in humans were more likely to harbor a dominant haplotype than infections in mosquitoes, potentially due to removal of unfit strains by the human immune system. Finally, within a given mosquito, there was little overlap in genetic composition of abdomen and head infections, suggesting that infections may be cleared from the abdomen during a mosquito’s lifespan. Taken together, our observations provide evidence for the mosquito vector acting as a reservoir of sequence diversity in malaria parasite populations.

## INTRODUCTION

Plasmodium falciparum has a complex life cycle that requires it to navigate multiple cellular and host transitions to sustain transmission. These include transitions both between human and mosquito hosts and between compartments within those hosts. In addition, distinct genotypes may be cotransmitted between hosts in a single bite or may accumulate within a host owing to serial superinfections. Such infections consisting of many different strains are particularly commonplace in highly endemic settings, such as some regions of sub-Saharan Africa (1), promoting both outcrossing in mosquito hosts and competition in human hosts. These factors, coupled with population bottlenecks and selective pressures encountered by P. falciparum throughout its life cycle, shape overall patterns of parasite genetic diversity and evenness (2).

Comparative population genetics of P. falciparum between the hosts and cellular environments through which the parasite transitions in natural cycles of transmission remains relatively unexplored. Several studies have compared markers of drug resistance loci between hosts, and an early report from Zambia observed very different allele frequencies in humans and mosquitoes (3, 4), suggesting differences in parasite population structure between hosts. However, subsequent reports from other settings using different genetic markers have not consistently observed this phenomenon (5, 6). As these studies used marker genes with few polymorphisms, analyses of individuals with complex coinfections were limited. While microsatellite markers overcome some of these limitations (7, 8), prior studies have not, to our knowledge, contrasted the genetic composition or diversity of highly polymorphic targets in naturally occurring infections of humans and mosquitoes that are participating in coincident transmission networks. By exploring these phenomena more closely, we can better understand what factors contribute to the diversity of malaria parasite populations.

We investigated variability in P. falciparum population genetic metrics across human and mosquito hosts in an area of high endemicity in Western Kenya. During a 14-month longitudinal cohort study, we detected P. falciparum parasites in human participants and in the heads and abdomens of resting Anopheline mosquitoes collected from their households (1). From each P. falciparum infection, we used amplicon deep sequencing of polymorphic segments of the parasite genes encoding apical membrane antigen 1 (*ama1*) and circumsporozoite protein (*csp*) to catalog complex P. falciparum infections in human blood, mosquito abdomens, and mosquito heads. We previously reported that parasite multiplicity of infection (MOI) as expressed by either marker was higher in mosquito abdomens harboring recently ingested parasites than in humans harboring blood-stage parasites (1). Due to this observation, and the robust immune defenses against P. falciparum in humans (9), we hypothesized that the mosquito host acts as a reservoir of sequence diversity of P. falciparum antigenic genes.

## RESULTS

### Data overview and analytic population.

Samples were collected over the course of 14 months (June 2017 to July 2018) from 38 households in three Kenyan villages. Mosquitoes were aspirated weekly from each household, and blood samples from household members were collected monthly. To the previously reported data on humans and mosquito abdomens (1), we added data from mosquito heads/thoraces (here denoted as heads). Over one-third of human samples (41.8%; 1,175/2,813) contained P. falciparum compared to 13.7% (199/1,448) of mosquito abdomens and 9.2% (134/1,462) of mosquito heads (Fig. S1 in the supplemental material). Of these, sequencing of at least one marker was successful in 86.2% (1,013/1,175) of human, 95.5% (190/199) of mosquito abdomen, and 98.5% (132/134) of mosquito head infections. Haplotype information from these 1,013 infections in 224 people and 322 infections in 244 mosquitoes constituted the analytic population.

10.1128/mbio.02277-22.1 FIG S1 Overview of sample infection status. Samples with and without P. falciparum infections, including what markers were sequenced for each infected sample. Download FIG S1, PDF file, 0.006 MB. Copyright © 2022 Lapp et al. 2022 Lapp et al. https://creativecommons.org/licenses/by/4.0/ This content is distributed under the terms of the Creative Commons Attribution 4.0 International license.

### Mosquito head infections are not a subset of their abdomen infections.

**FIG 1:**
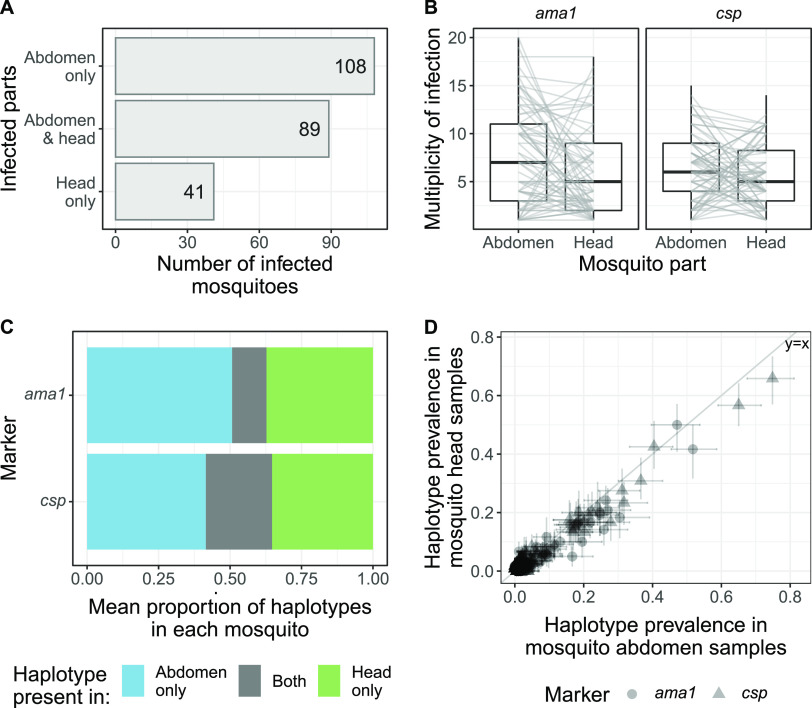
Mosquito abdomens and heads do not contain similar infections. (A) P. falciparum infection of mosquito abdomens and heads of mosquitoes for which both compartments were tested using PCR. (B) Multiplicity of infection of infected mosquito abdomen and head samples. Gray lines link MOIs from an individual mosquito. (C) For 89 mosquitoes with infections of both the abdomen and the head, the proportions of the set of haplotypes in the mosquito found in the abdomen only, head only, or both are shown. The mean counts for each of the three groups were used to obtain the proportions. (D) Prevalence of each *ama1* and *csp* haplotype in the mosquito abdomen population compared to the mosquito head population. Each dot represents a unique *ama1* or *csp* haplotype, and bars indicate the 95% bootstrapped confidence intervals.

Parasite development within the mosquito host begins in the abdomen, following which sporozoites must traverse the midgut wall to reach the salivary glands in the head; however, it is not known how quick and comprehensive this egress is. We hypothesized that if both midgut and salivary gland infections persist throughout the mosquito’s lifespan (i.e., incomplete egress from the midgut), haplotypes in a mosquito’s head would be a subset of those in the abdomen. Among mosquitoes in which at least one compartment was infected, P. falciparum was detected in both the abdomen and the head in 89/238 (37.4%), in only the abdomen in 108/238 (45.4%), and in only the head in 41/238 (17.2%) (Fig. 1A). The latter finding suggests that infections may be completely cleared from the abdomen within the span of a mosquito’s lifetime.

We next compared the haplotype compositions of infections in the 89 mosquitoes in which P. falciparum was detected in both the head and the abdomen. Median multiplicity of infection (MOI) was generally higher in the abdomen (*csp* = 6; *ama* = 7) than in the head (*csp* = 5; *ama* = 5), but this trend was not consistent at the individual mosquito level (Fig. 1B). We calculated the percentage of *ama1* or *csp* haplotypes found only in the head or the abdomen or observed in both compartments (i.e., the Jaccard distance; intersect/union) within each mosquito. While some haplotypes were observed in both compartments of a given mosquito (mean for *ama1* = 12.0% and *csp* = 23.7%), the majority of haplotypes were either private to the abdomen (mean for *ama1* = 50.7% and *csp* = 41.5%) or head (mean for *ama1* = 37.3% and *csp* = 34.8%) (Fig. 1C and Fig. S2A and B). Despite this limited overlap, sharing between abdomens and heads from the same mosquito was higher than sharing between random pairs of abdomens and heads (Fig. S2C; Kolmogorov-Smirnov test, *P* ≤ 1 × 10^−10^ for both markers). Additionally, we observed no difference in haplotype overlap between abdomens and heads from mosquitoes who had recently fed and those who had not (Fig. S2D; both Wilcoxon rank-sum tests, *P* > 0.2).

10.1128/mbio.02277-22.2 FIG S2 Summary of haplotypes found in the mosquito abdomen, mosquito head, or both compartments. (A) Sample-level counts. (B) Jaccard distance of haplotypes in abdomens and heads from the same mosquito calculated as the intersect over the union of haplotypes in each mosquito. Color indicates the total number of haplotypes present in the mosquito or the size of the union. (C) Jaccard distance of haplotypes observed in abdomens and heads compared between the same mosquito and different mosquitoes. The point is the median value. (D) Jaccard distance for mosquitoes who had not recently fed (unfed and gravid) compared to those who had recently fed (blood fed or half gravid). Download FIG S2, PDF file, 0.04 MB. Copyright © 2022 Lapp et al. 2022 Lapp et al. https://creativecommons.org/licenses/by/4.0/ This content is distributed under the terms of the Creative Commons Attribution 4.0 International license.

To determine whether the differences in haplotype composition between abdomen and head infections within a single mosquito corresponded to differences at the host population level, we compared between mosquito compartments haplotype population-level prevalences, defined as the number of samples in which a haplotype was observed. Both *ama1* and *csp* haplotype prevalences were similar between mosquito abdomen and head populations (Fig. 1D), suggesting that the transition from oocyst to sporozoite does not alter the diversity of circulating parasites. Owing to this population-level similarity in prevalences and our observation that abdomen and head parasite populations from the same mosquito appear to frequently represent different infections, we subsequently performed all comparisons between the two P. falciparum hosts humans and mosquitoes, where mosquito samples included both abdomen and head samples.

### The P. falciparum population in mosquitoes is more diverse and more even than the population in humans.

**FIG 2:**
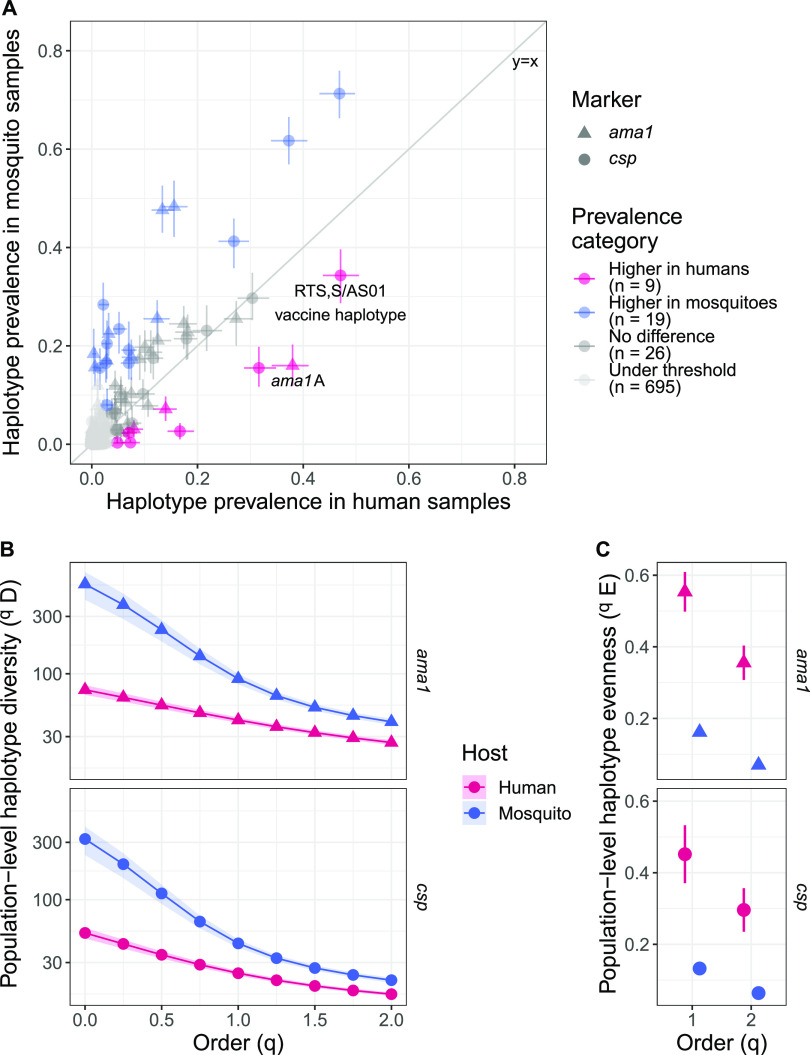
The mosquito P. falciparum population is more diverse and less even than the human P. falciparum population. (A) Prevalence of each *ama1* and *csp* haplotype in the human population compared to the mosquito population. Each dot represents a unique *ama1* or *csp* haplotype, and bars indicate the 95% bootstrapped confidence intervals. The lower threshold was defined as haplotypes observed in fewer than 5% of combined human and mosquito samples. Haplotype prevalences were considered higher in one compartment if the 95% bootstrapped confidence intervals did not overlap the expected prevalence (i.e., the overall prevalence across all samples). (B) Diversity of P. falciparum populations by host and genetic marker across orders of diversity. Ribbons are bootstrapped 95% confidence intervals. Higher values indicate more diversity. The slope of the line across orders *q* is a measure of haplotype evenness in the population. (C) Haplotype evenness of human and mosquito samples. Bars are bootstrapped 95% confidence intervals. Higher values indicate more similar prevalence of haplotypes in the population.

To investigate signatures of differential bottlenecks or selection during parasite transition between mosquito and human hosts, we compared population-level differences in parasite haplotype prevalence among mosquitoes and humans, where differences in prevalence may indicate differential bottlenecks or selection. Across all infections, we observed high haplotype richness, with 456 *ama1* and 298 *csp* distinct haplotypes. The vast majority of these were low-frequency haplotypes, many of which were observed in only one host (Fig. S3 and S4). Among 54 distinct haplotypes (both *ama1* and *csp*) with a prevalence above 5% across all samples, we observed 28 haplotypes with differential prevalence across hosts; 19 were more common in mosquito infections, and 9 were more common in human infections (Fig. 2A), consistent with our observation of higher average mosquito MOIs (1) (Fig. S5).

10.1128/mbio.02277-22.3 FIG S3 Haplotype richness and evenness across host compartments. Mean proportion of haplotypes present in each compartment expressed as haplotype richness (left, including each unique haplotype once) and sample haplotype prevalence (right, counting the number of times each haplotype was observed). Download FIG S3, PDF file, 0.006 MB. Copyright © 2022 Lapp et al. 2022 Lapp et al. https://creativecommons.org/licenses/by/4.0/ This content is distributed under the terms of the Creative Commons Attribution 4.0 International license.

10.1128/mbio.02277-22.4 FIG S4 Randomized minimum spanning trees for nucleotide haplotype sequences. Download FIG S4, PDF file, 0.1 MB. Copyright © 2022 Lapp et al. 2022 Lapp et al. https://creativecommons.org/licenses/by/4.0/ This content is distributed under the terms of the Creative Commons Attribution 4.0 International license.

10.1128/mbio.02277-22.5 FIG S5 Within-sample diversity. When *q* = 0, the within-sample diversity is equivalent to the multiplicity of infection. Download FIG S5, PDF file, 0.02 MB. Copyright © 2022 Lapp et al. 2022 Lapp et al. https://creativecommons.org/licenses/by/4.0/ This content is distributed under the terms of the Creative Commons Attribution 4.0 International license.

We next used haplotype prevalence to quantify population-level diversity across orders of diversity (*q*) ranging from equal weight to each haplotype (*q *= 0, equivalent to haplotype richness or the number of distinct haplotypes observed) to downweighing rare haplotypes (*q* = 2, effective number of highly abundant haplotypes) (10). The mosquito parasite population was more diverse than the parasite population in human hosts (Fig. 2B and Fig. S6A). This trend is consistent even when accounting for differences in sample size between hosts, multiple samples per host, different sampling schemes between hosts, potential differences between symptomatic and asymptomatic infections, differences in MOI, haplotypes with rare variants, and limitations of using estimated diversity (Fig. S6B) as well as across villages (Fig. S6C) and transmission seasons (Fig. S6D). To determine whether there may be differences in the changes in diversity during the transition between different parasite host compartments, we also compared separately the diversity of the parasite populations of humans, mosquito abdomens, and mosquito heads (Fig. S6E). The parasite populations of mosquito abdomens and heads were relatively similar, and the human parasite population was more diverse than the parasite population in both mosquito abdomens and heads. Moreover, as evidenced by the steeper decline in diversity with increasing *q* in mosquitoes than that observed in humans (Fig. 2B), haplotype prevalences were more uneven in the parasite population of the mosquito (Fig. 2C), particularly in the head (Fig. S7A). These trends were generally consistent, although not always significant, when performing the same sensitivity analyses as for the diversity comparison (Fig. S7B to D). This indicates that mosquitoes contained a larger relative number of infrequent haplotypes than humans. Even so, higher diversity in the mosquito host is still apparent when downweighing the contribution of these minor haplotypes. Taken together, these results indicate that there may be a greater relative loss in diversity across the transition from mosquitoes to humans than from humans to mosquitoes.

10.1128/mbio.02277-22.6 FIG S6 Population-level diversity sensitivity analyses. (A) Rarefaction curves for various orders of diversity (*q*). True (asymptotic) diversity can be calculated after the rarefaction curve flattens out; otherwise, the computed true diversity is a lower bound. Comparisons can be made for true diversity at orders of diversity above *q* = 1 in our dataset because the human diversity curve flattens out and is lower than the mosquito diversity curve (which is a minimum bound on diversity). (B) Sensitivity analyses comparing diversity between humans and mosquitoes for subsampled data. (C) Sensitivity analysis comparing diversity across villages. (D) Sensitivity analysis comparing diversity across seasons. The high season is considered to be April to September. High 1 is the first high season, and high 2 is the second high season. (E) Diversity of parasites in mosquito abdomens and mosquito heads compared to that in humans and combined mosquito samples. Download FIG S6, PDF file, 0.05 MB. Copyright © 2022 Lapp et al. 2022 Lapp et al. https://creativecommons.org/licenses/by/4.0/ This content is distributed under the terms of the Creative Commons Attribution 4.0 International license.

10.1128/mbio.02277-22.7 FIG S7 Population-level evenness sensitivity analyses. (A) Evenness of parasites in mosquito abdomens and mosquito heads compared to that in humans and combined mosquito samples. (B) Sensitivity analyses comparing evenness between humans and mosquitoes for subsampled data. Evenness based on the asymptotic calculation is much more similar between humans and mosquitoes; however, this method assumes that we have fully captured the diversity of both populations, which is unlikely due to the high prevalence of P. falciparum in the region. (C) Sensitivity analysis comparing evenness across villages. (D) Sensitivity analysis comparing evenness across seasons. Download FIG S7, PDF file, 0.01 MB. Copyright © 2022 Lapp et al. 2022 Lapp et al. https://creativecommons.org/licenses/by/4.0/ This content is distributed under the terms of the Creative Commons Attribution 4.0 International license.

### Dominant haplotypes within infections are more common in humans than in mosquitoes.

**FIG 3:**
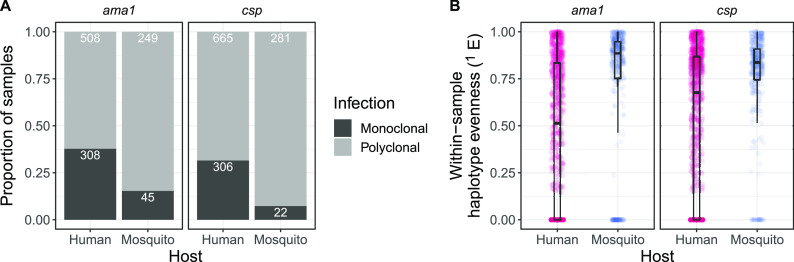
Compared to mosquito samples, human samples are more often dominated by a single haplotype. (A) Proportion of samples with monoclonal and polyclonal infections. Numbers are counts for each category. (B) Distributions of within-sample evenness (*q* = 1) by genetic marker and host. Lower values indicate more dominance by individual haplotypes within the strain mixture.

In addition to lower population-level diversity in humans than in mosquitoes, we also observed lower within-sample diversity (Fig. S5) and proportionately more monoclonal infections in humans (Fig. 3A; both Fisher’s exact test, *P* < 1 × 10^−12^). To further investigate whether human infections are more often dominated by one or a few haplotypes than mosquito infections, we calculated the haplotype evenness for each infection, which examines the haplotype abundance within an infection based on sequencing reads. A lower value indicates that the infection consists of mostly reads from a single haplotype or, in other words, is dominated by a majority haplotype. Median evenness values for mosquito infections were higher (*ama1* = 0.88; *csp* = 0.84) than those for human infections (*ama1* = 0.51; *csp* = 0.67; all *P* < 1 × 10^−4^) (Fig. 3B). This observation was robust to taking the maximum evenness among the two markers and to differences in haplotype filtering (all *P* < 1 × 10^−10^) (Fig. S8). This differential composition of polyclonal P. falciparum infections between hosts supports a differential in selective landscapes that may further enable the preservation of diverse P. falciparum populations in Anopheline mosquitoes.

10.1128/mbio.02277-22.8 FIG S8 Haplotype evenness sensitivity analyses. (A) Taking the maximum evenness value between *ama1* and *csp*. (B) For precensored haplotype read counts. Both sensitivity analyses show the same trend as Fig. 3B in the main text. Download FIG S8, PDF file, 0.2 MB. Copyright © 2022 Lapp et al. 2022 Lapp et al. https://creativecommons.org/licenses/by/4.0/ This content is distributed under the terms of the Creative Commons Attribution 4.0 International license.

## DISCUSSION

We compared P. falciparum genetic diversity across several host compartments that the parasite must successfully navigate to sustain transmission. Parasite genetic diversity was increased relative to humans during the mosquito stages. In addition, individual infections were composed differently in mosquitoes and humans, with human infections more commonly harboring dominant members. Collectively, our observations suggest that mosquito-stage infections not only maintain diversity in P. falciparum populations through recombination but also act as a reservoir of parasite sequence diversity.

We observed, using multiple metrics, more parasite genetic diversity in mosquitoes than in humans. This high diversity contrasts with the known marked reduction in parasite biomass during the transition from the human to the mosquito abdomen (11), which might be expected to constrain parasite diversity. One potential explanation for this is the possibility of cryptic genotypes in humans undetected by marker sequencing; this has been reported in experimental studies (7), although the large range of MOIs we observed in humans suggests that these infections were not systematically undersampled. Alternatively, the reduced diversity in humans could result from large reductions in population size and negative selective pressures as the parasite passes from mosquitoes to the human liver and into the blood stage. Mosquitoes are the location of parasite sexual recombination and therefore certainly provide a site for genomic diversification, but this seems unsuited to explain the diversity of these short segments in *ama1* and *csp* that do not harbor known recombination hot spots (12). A probable contributor to this high mosquito diversity is multiple or interrupted feeds on infected hosts, which would allow strains to accumulate in the mosquito abdomen. This feeding behavior has been reported for Anopheles gambiae (13), may be enhanced by human P. falciparum infection (14), and may increase the likelihood of subsequent infections (15). Additionally, P. falciparum adaptation to evade the immune system of local *Anopheles* strains (16), as well as imposition of selective pressures on the *Anopheles* vector to the parasites’ benefit (17), may reduce differences in fitness between distinct parasite strains within the mosquito and lead to an accumulation of genetic diversity at the population level. However, some of these novel strains may be unfit to survive the human host. Indeed, prior work on arbovirus infection found an accumulation of mutations in mosquitoes that led to fitness costs during vertebrate infection (18). Despite these plausible explanations for constrained diversity in humans and higher diversity in mosquitoes, the mechanism by which mosquitoes maintain such high parasite diversity when their parasite population is necessarily sampled from the less diverse human population remains to be fully elucidated.

Within individual infections, we observed higher dominance of haplotypes in human infections than in mosquito infections, while on a larger scale, the P. falciparum haplotype population was more evenly distributed among humans than among mosquitoes. These differences may result from the differential selection landscapes between hosts, in particular for the proteins encoded by our gene targets AMA1 and CSP, which harbor epitopes that are known targets of functional human immunity (19). In humans, the concurrent maintenance in the population of multiple viable alleles due to balancing selection, paired with the removal of deleterious alleles due to negative selection, could produce a relatively high evenness of haplotypes in the human parasite population even as individual infections are shaped by directional selection resulting from individual host immune responses. In contrast, the relative lack of differential fitness in the mosquito host described above may lead to even parasite strain abundances within a mosquito.

Comparison of paired abdomens and heads from the same mosquito revealed striking differences between P. falciparum presence and haplotype composition. As expected, given the delay between midgut and salivary gland infections, many mosquitoes had haplotypes private to the abdomen that were not present in the head. More surprising was the observation of mosquitoes with haplotypes private to the head that were absent from the abdomen, suggesting that infections do not reliably persist in a mosquito’s abdomen throughout its lifespan. While these differences may again be due to cryptic haplotypes, the identification of mosquitoes with infections in the head but not the abdomen using sensitive PCR detection methods (20, 21) indicates that cryptic haplotypes likely cannot explain all of the observed differences. Despite these discrepancies between abdomens and heads from a given mosquito, at the population level, haplotype composition and diversity were similar between mosquito abdomens and heads, suggesting that the selective pressures for or against certain haplotypes (or lack thereof) may be similar in these two compartments.

Our findings highlight the role of the mosquito host in harboring sequence diversity of P. falciparum parasites. A unique feature of genetic diversity in P. falciparum compared to other organisms is the preponderance of low-frequency alleles (22). A prior modeling study suggested that this phenomenon may be the result of the complex, “unconventional” life cycle of P. falciparum, specifically the bottlenecks and host transitions that intensify both random genetic drift as well as natural selection (2). Consistent with this, we observed many haplotypes private to one host, which was more prominent in mosquitoes. As noted above, meiotic recombination is unlikely to be the main contributor to the diversity we cataloged, and the mechanisms by which these low-frequency and private alleles arise remain obscure. However, our observations furnish compelling evidence for a role of the mosquito vector in accumulating genetic diversity in genic regions likely not under positive selection in mosquitoes. While it appears that the majority of these polymorphisms tend to be selected against in humans, the diversity in mosquitoes nevertheless acts as a continual supply of novel alleles and allelic combinations that may by chance be advantageous and exploited by the parasite during human infection.

This study has limitations. First, the inability to sample parasites from mosquitoes without sacrificing them precludes a comprehensive study of paired mosquito abdomen and head infections over time. Even so, we were still able to identify similarities and differences between the haplotype populations in these two compartments. Second, parasites accumulate in mosquitos over multiple blood meals, which presumably increases diversity in mosquitoes; however, this phenomenon is likely balanced by parasite accumulation in humans owing to superinfection. Third, the mosquito and human sampling schemes were different, potentially biasing sampling comprehensiveness between hosts. To mitigate the risk that this potential imbalance influenced our results, we performed comparative population analyses using empirical methods with a fixed coverage threshold (10) and sensitivity analyses. Fourth, many of the human and mosquito infections had very low parasite densities, which not only increases the possibility of failing to detect infections but also increases the possibility of false haplotype discovery (23). To reduce the inclusion of false haplotypes to the greatest extent possible, we performed strict haplotype censoring to remove potential false positives (1) and performed sensitivity analyses on key findings to determine whether haplotype filtering criteria influenced the results.

In conclusion, our comparison of P. falciparum haplotypes observed in natural, coincident infections of humans, mosquito abdomens, and mosquito heads revealed greater genetic diversity in mosquitos than in human populations and infections. This provides evidence for the role of the mosquito vector in harboring sequence diversity of the malaria parasite population.

## MATERIALS AND METHODS

### Ethics statement.

All adults and parents or legal guardians for individuals under 18 years old provided written informed consent. Children over 8 years old also provided verbal assent. The study was approved by the ethical review boards of Moi University (2017/36) and Duke University (Pro00082000).

### Study design and sampling.

The study design and sample processing have been described previously (1). Briefly, a longitudinal cohort of participants (1 year of age or older) residing in 38 households in three villages in Western Kenya were followed from June 2017 to July 2018. For each participant, dried blood spots (DBS) were collected monthly and at any time participants had malaria symptoms. One morning each week, indoor resting mosquitoes were collected from participant households using vacuum aspiration, and, following morphologic identification, the abdomen was separated from the head and thorax of female *Anopheles* mosquitoes. Genomic DNA was isolated from DBS, mosquito abdomens, and mosquito heads/thoraces; P. falciparum was detected in these extracts using a real-time PCR assay. Segments of approximately 300 nucleotides of the P. falciparum *ama1* and *csp* genes were amplified and sequenced on an Illumina MiSeq platform. Negative controls were included for all PCR amplification assays; all negative controls were negative. Haplotype inference was performed using DADA2 v1.8 (24) with custom read and haplotype filtering as described in Sumner et al. (1). Briefly, haplotypes were removed if they were supported by fewer than 250 reads or fewer than 3% of the total number of reads for a sample, deviated from the expected length, were a minority haplotype differing by only one variant position from a majority haplotype present with greater than 8 times the read depth, or contained a variant position only variable within that haplotype.

The output was a set of quality-filtered *ama1* and *csp* reads and corresponding parasite haplotypes for each P. falciparum infection. Sequencing was only possible at two targets because of the combination of a very small amount of material for each sample and very low infection density for many samples.

We performed parallel analyses of amplicon deep-sequenced segments of the P. falciparum *ama1* and *csp* marker genes. Since *ama1* and *csp* are unlinked markers found on different chromosomes, to some extent, these parallel analyses can be considered pseudoreplicates, where similar results for both markers increase confidence in our findings.

### Within-mosquito comparison.

For each mosquito with a P. falciparum infection in both the abdomen and the head, the Jaccard distance (25) was calculated for the haplotypes in the abdomen-head pair,

(1) $$J\left(H_{a},\;H_{h}\right)=|\frac{H_{a}\cap H_{h}}{H_{a}\cup H_{h}}|$$

where *H_a_* is the set of haplotypes in the abdomen, and *H_h_* is the set of haplotypes in the head.

### Haplotype prevalence.

For each haplotype, population-level prevalence was determined for 5 distinct populations: the entire sample set, all human samples, all mosquito samples, mosquito abdomens, and mosquito heads. Prevalence was calculated as the proportion of samples harboring that haplotype; 95% confidence intervals were computed from 100 bootstrapped data sets. Haplotypes were considered low frequency if they occurred in fewer than 5% of all samples. Haplotypes that were not low frequency (i.e., above a threshold of 5% prevalence) were considered higher in a given compartment if the range of bootstrapped prevalences did not overlap the expected prevalence (i.e., the overall prevalence across all samples).

### Randomized minimum spanning trees.

To visualize the relatedness of haplotypes, we calculated pairwise distances using the dist.dna() function in the R package ape v5.6-2 (26) with the K80 evolutionary model, computed randomized minimum spanning trees (27) using the rmst() function in pegas v1.1 (28), and visualized the trees in ggtree v3.0.4 (29).

### Diversity and evenness.

For analyses between humans and mosquitoes, all mosquito abdomen and head samples were considered mosquito samples, providing a maximum of 2 samples from each mosquito. All human samples were included unless otherwise noted, as individuals may acquire additional infections over time and the relative abundance of coinfecting strains may change over time.

**(i) Population level.** For the set of mosquito samples and the set of human samples, we calculated the population-level diversity of haplotypes, rarefaction curves, and population evenness using the R packages iNEXT.4steps v1.0.1 (10) and iNEXT.3D v1.0.1 (30).

Diversity was calculated using the following equation (10, 31):

(2) Dq=(∑i=1npiq)1/(1−q)

where *q* is the order of diversity, *n* is the number of distinct haplotypes, and *p_i_* is the prevalence of haplotype *i* in the sample set. *D* was computed across a range of values *q* between 0 and 2, where higher numbers correspond to upweighting haplotypes that are more abundant in the overall population. True diversity was not accurately calculable for low orders of diversity (*q *< 1) due to an abundance of unsampled rare haplotypes. Therefore, to enable comparison of diversity between the human and mosquito haplotype populations, we calculated the empirical diversity at a standardized coverage of the host population’s haplotypes (90.1% for *ama1* and 94.7% for *csp*).

Because haplotypes observed within multiple samples from a single individual may either be correlated or more easily detectable owing to repeated testing, we performed a sensitivity analysis in which we included a single sample from each individual (i.e., one sample from each mosquito and one sample from each person). Additional sensitivity analyses were performed (i) by using true (asymptotic) diversity (for *q *≥ 1), (ii) by using a subsampled data set including the same number of human and mosquito samples, (iii) by using a subsampled data set including the same number of host samples per week (to account for differences in mosquito and human sampling schemes), (iv) by using a data set excluding samples from symptomatic humans to reduce the possibility of undetected cryptic haplotypes that may be present in high-density symptomatic infections, (v) by defining *p_i_* as the frequency of haplotypes in the total set of haplotypes (to account for differences in MOI between infections), and (vi) by using a data set including only haplotypes with variation at amino acid positions that are variable in both hosts (to limit potential false positives by using this stricter set of haplotype-filtering criteria). We also calculated diversity by village and season (we defined high season as April to September, inclusive).

We calculated haplotype evenness using the following equation (32):

(3) Eq= Dq−1H−1

where *H* is the haplotype richness or the number of distinct haplotypes in the population. For *q *= 0, evenness is defined as 1, and for *H *= 1, evenness is defined as 0. The same sensitivity analyses described above for diversity were performed for evenness.

**(ii) Within sample**. For each sample, we computed haplotype diversity and evenness using equations 1 and 2. In this case, *p_i_* in equation 1 is the relative read abundance of each haplotype, *^q^ D* is the within-host diversity, and *H* is the MOI of the infection.

To compare evenness between human and mosquito hosts, we computed a zero-one inflated Beta regression model using the R package gamlss v5.4-1 (33) with host as the main exposure, evenness as the outcome, log_2_-transformed haplotype reads as a covariate, and individual as a random effect. To determine whether incorporating information from both markers influenced differences in evenness between hosts, for each sample, we selected the highest evenness value (between *ama1* and *csp*) and compared these values between humans and mosquitoes. Finally, to explore if evenness values were biased by the initial enforcement of haplotype quality-filtering criteria that were partially based on within-sample haplotype proportion, we performed a sensitivity analysis using unfiltered haplotypes. These haplotypes were inferred by DADA2 v1.8 (24) from input reads that passed upstream read quality filtering. Using these unfiltered haplotypes, we used the same methods as above to compute and compare evenness.

### Data analysis and visualization.

Comparison across groups was performed using Wilcoxon rank-sum tests, Fisher’s exact tests, or Kolmogorov-Smirnov tests. All data analyses and visualization were performed in R v4.1.1 (34) and RStudio v2021.9.0.351 (https://www.rstudio.com/) using the following packages: tidyverse v1.3.1 (35), ape v5.6-2 (26), cowplot v1.1.1 (36), scales v1.1.1 (37), and ggtext v0.1.1 (38).

### Data availability.

Raw sequencing reads are available from NCBI (BioProject PRJNA646940) (1, 39). All processed data and code to reproduce the analyses and figures can be found on GitHub (40).
