# Supplementary material for: Plasmodium falciparum Genetic Diversity in Coincident Human and Mosquito Hosts
Source: mBio. 2022 Sep 8;13(5):e02277-22. doi: 10.1128/mbio.02277-22 (PMC9600619; doi:10.1128/mbio.02277-22)
Supplement: FIG S1 [file mbio.02277-22-s0001.pdf]

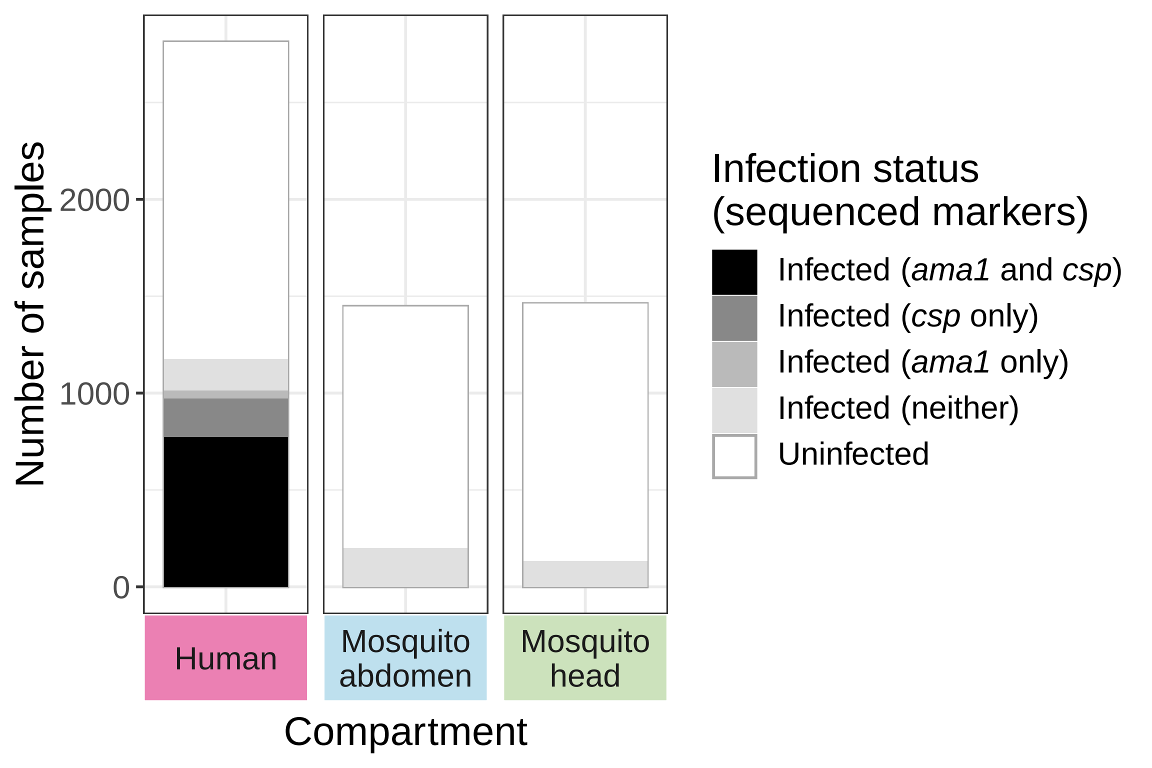
Number of samples
2000
1000
0
Human
Mosquito abdomen
Mosquito head
Compartment
Infection status (sequenced markers)
Infected (*ama1* and *csp*)
Infected (*csp* only)
Infected (*ama1* only)
Infected (neither)
Uninfected
