# Supplementary figures and images for: Plasmodium falciparum Genetic Diversity in Coincident Human and Mosquito Hosts
Source: mBio. 2022 Sep 8;13(5):e02277-22. doi: 10.1128/mbio.02277-22 (PMC9600619; doi:10.1128/mbio.02277-22)

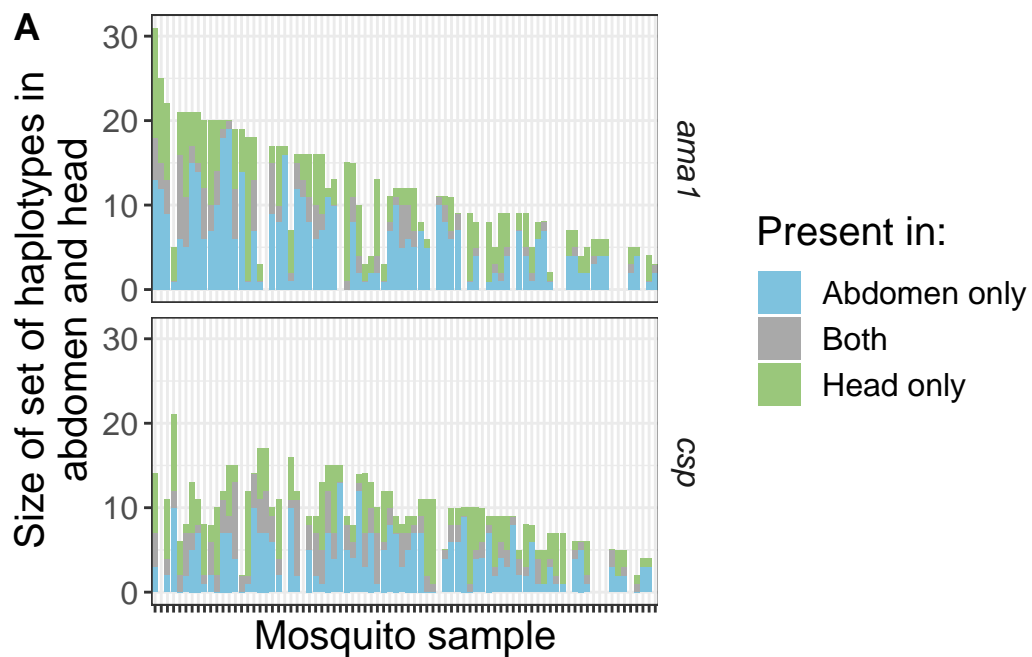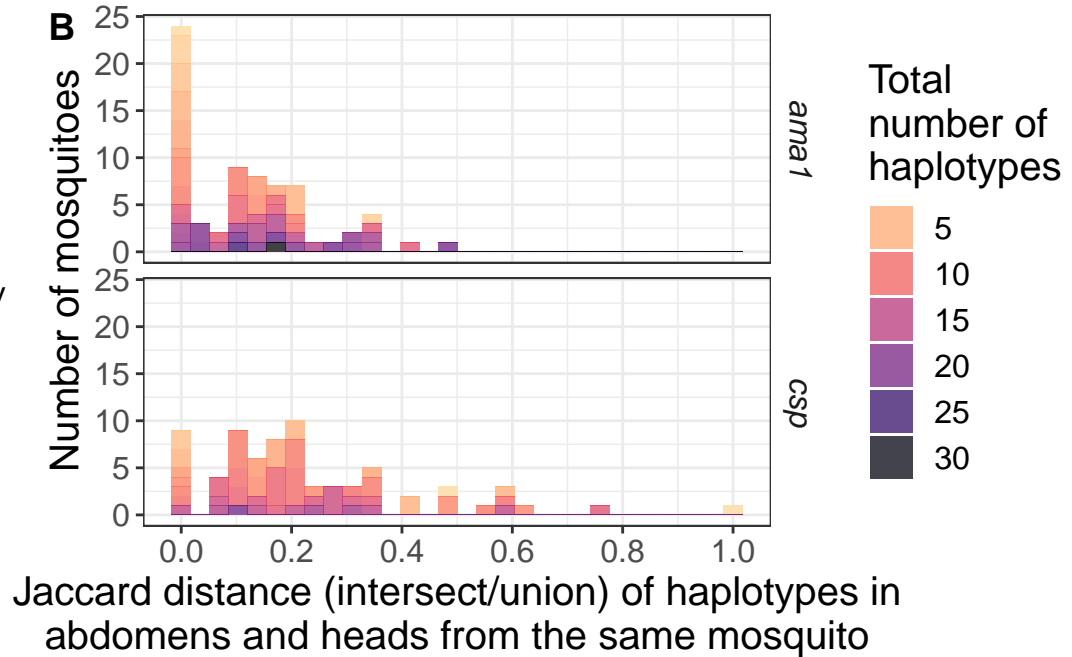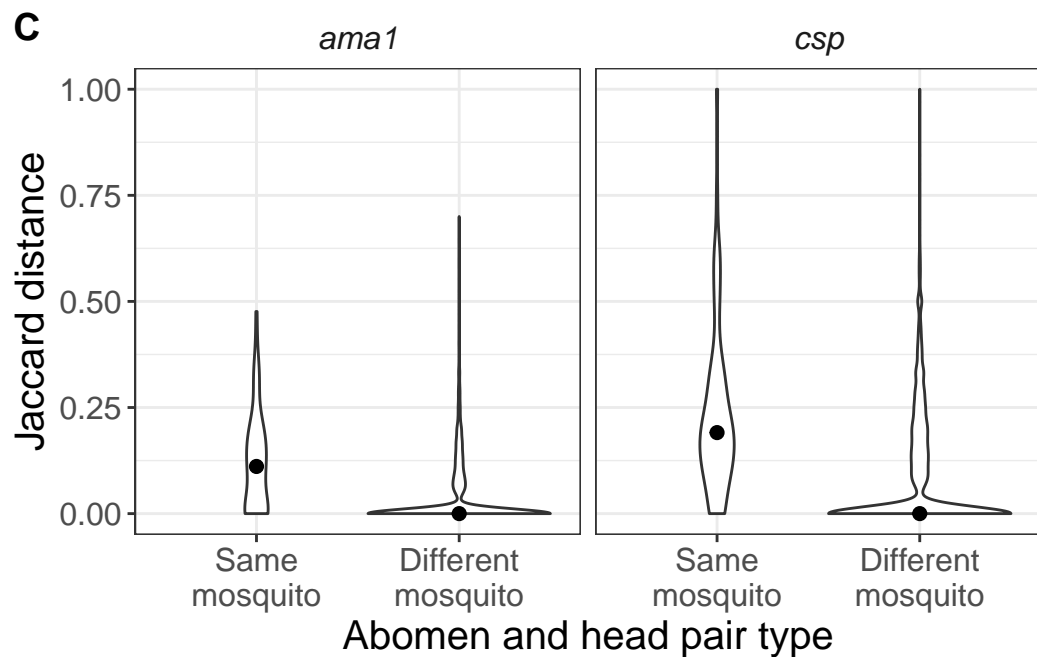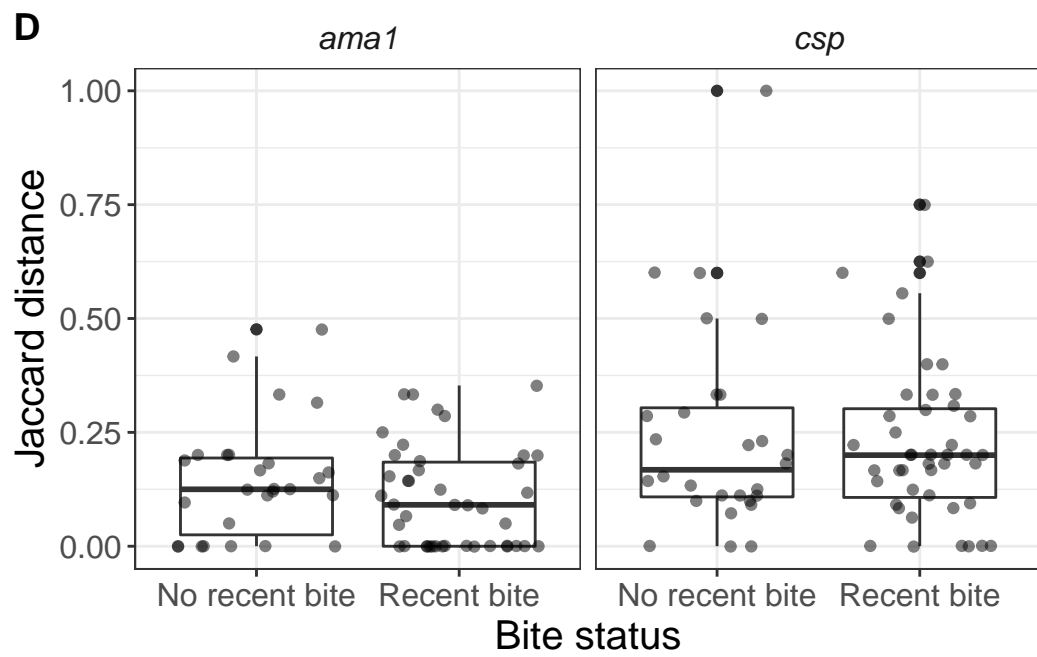

Supplement: FIG S2 [file mbio.02277-22-s0002.pdf]

Relative haplotype abundance

Haplotype  
richness

Haplotype  
prevalence

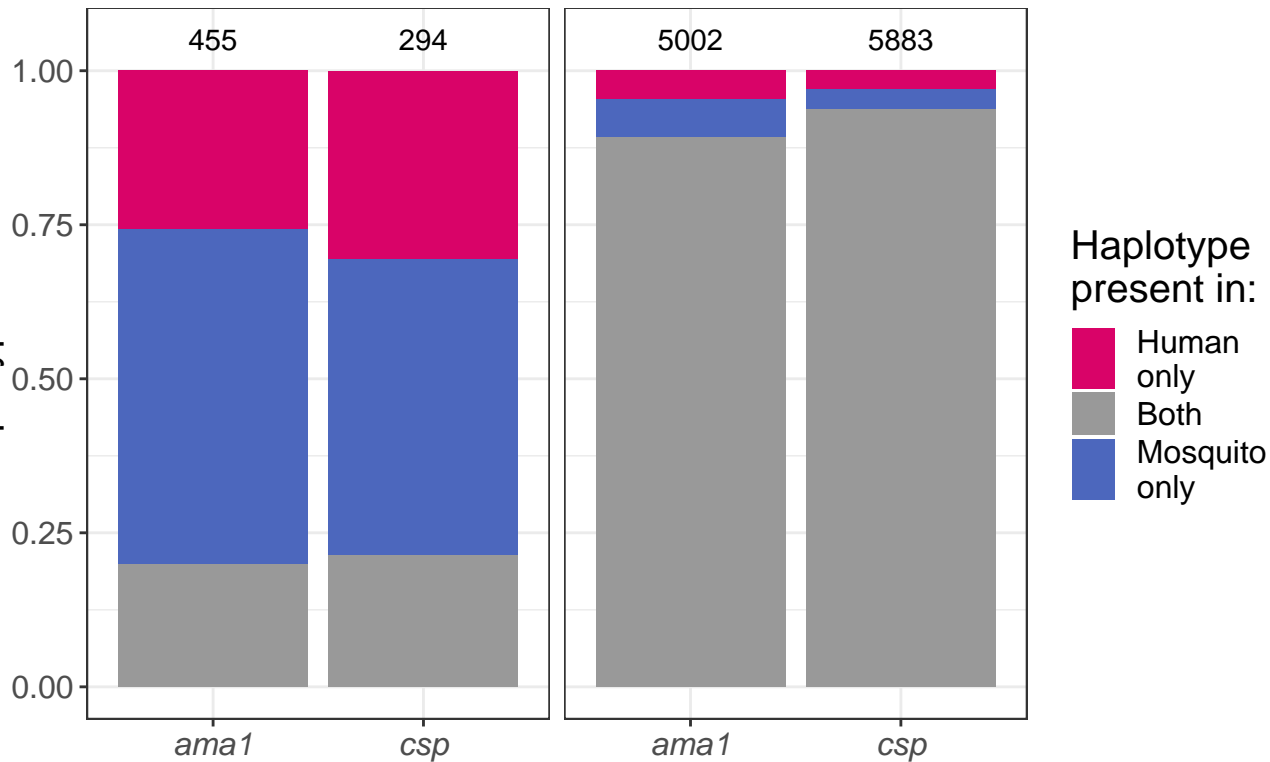

Supplement: FIG S3 [file mbio.02277-22-s0003.pdf]

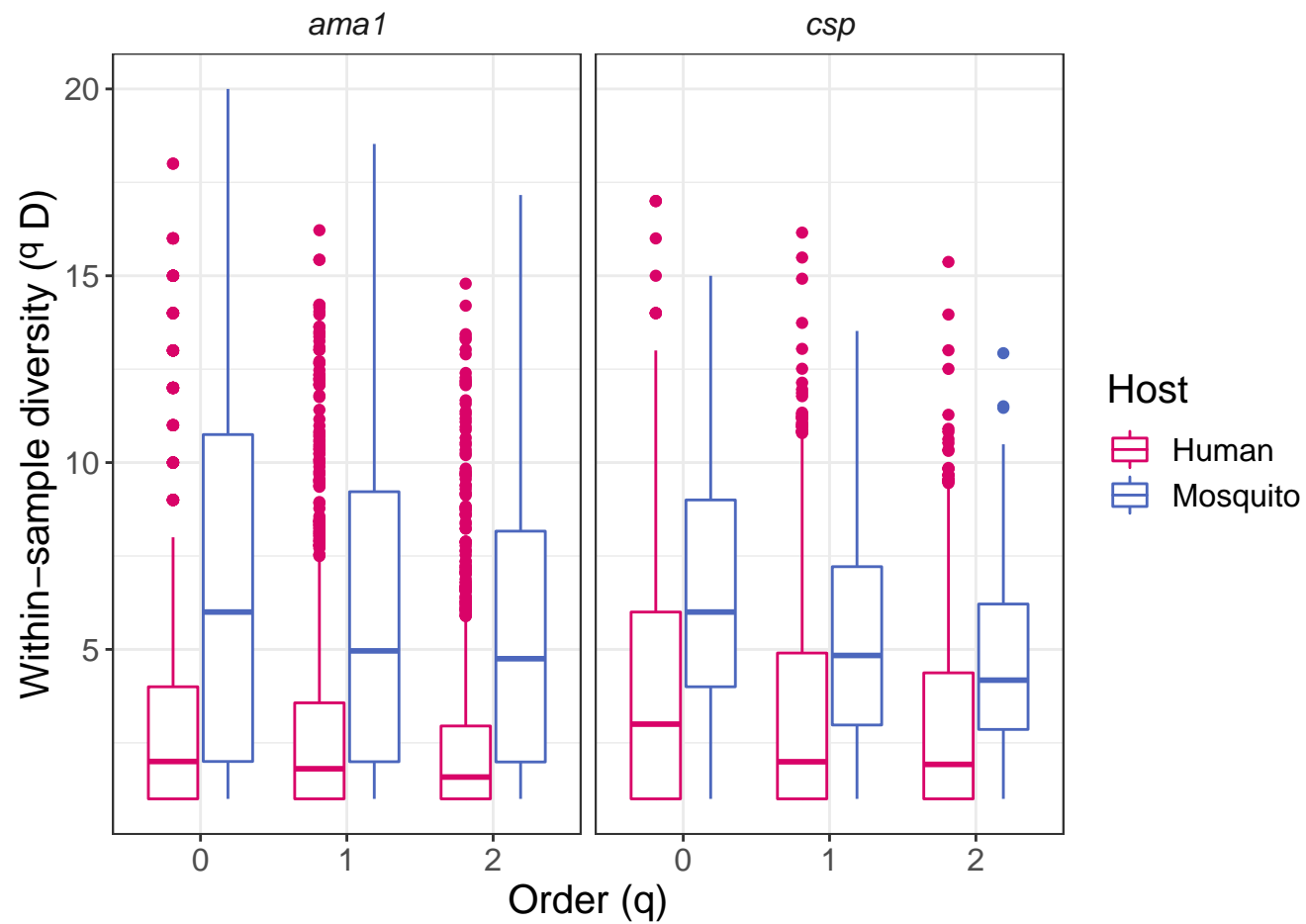

Supplement: FIG S5 [file mbio.02277-22-s0005.pdf]

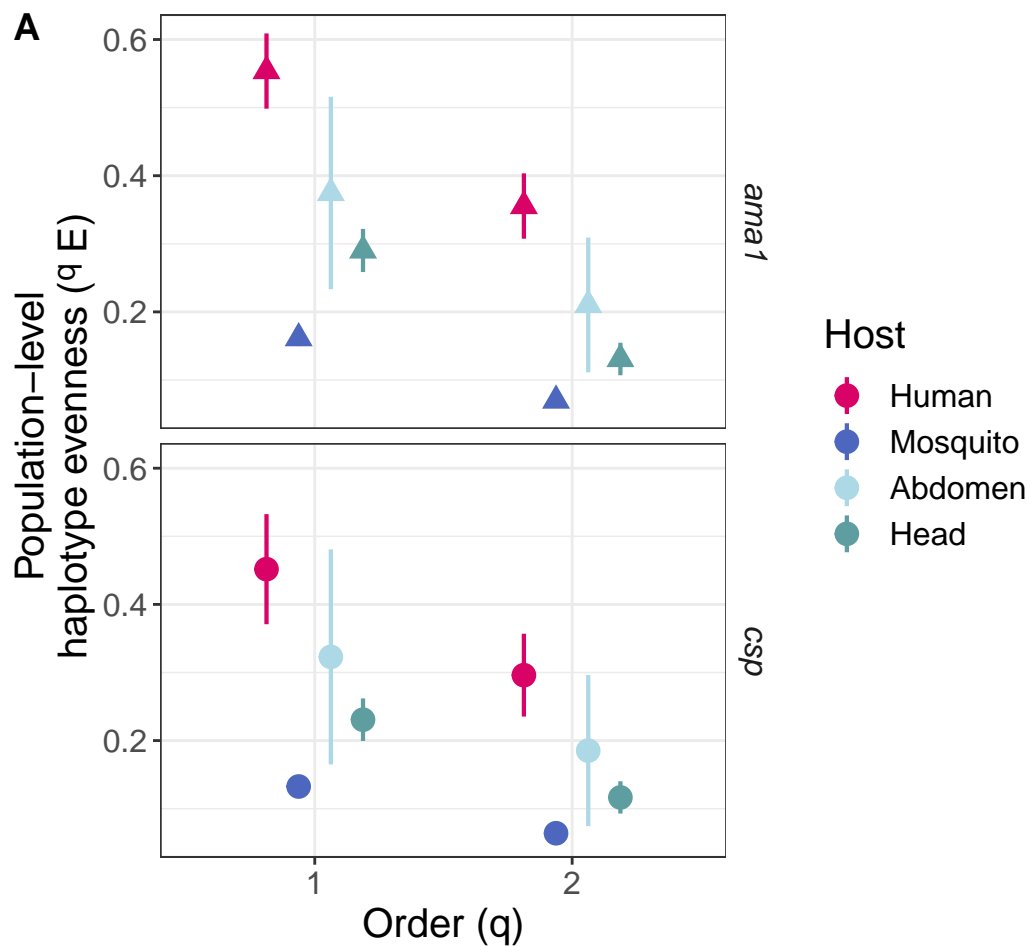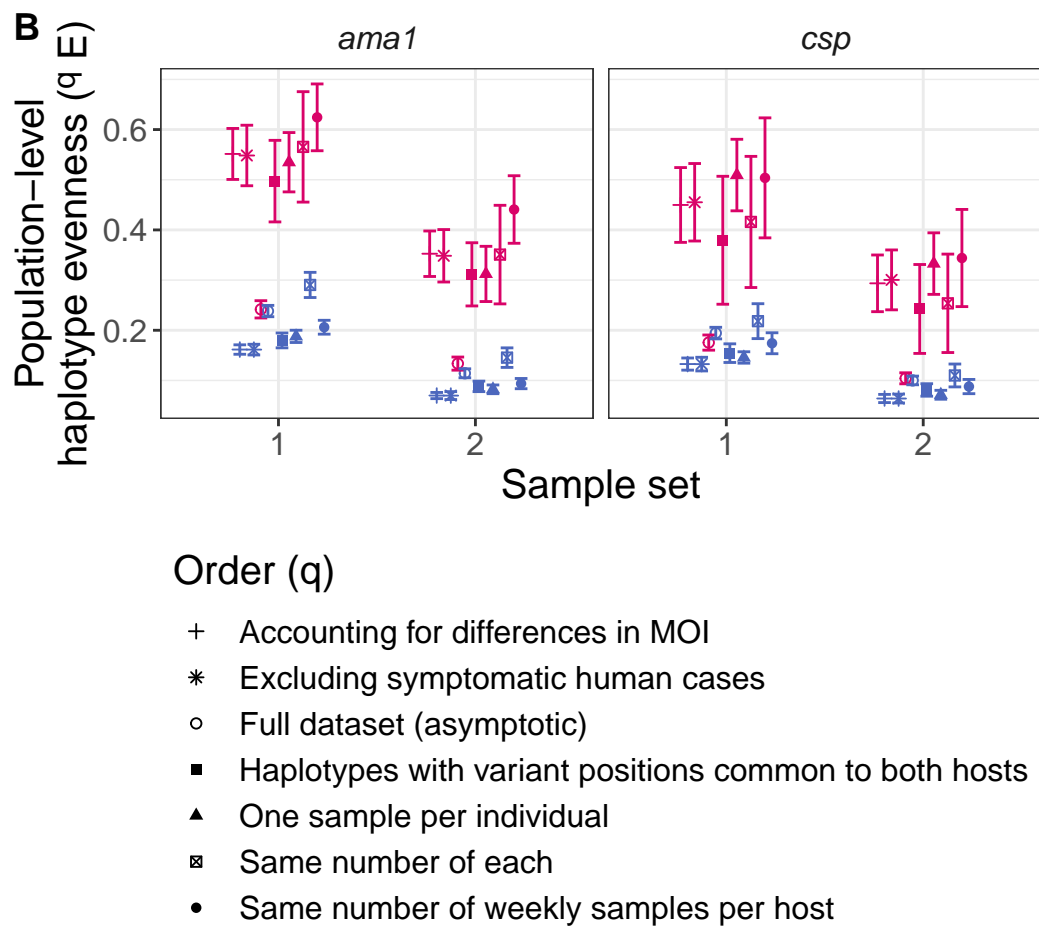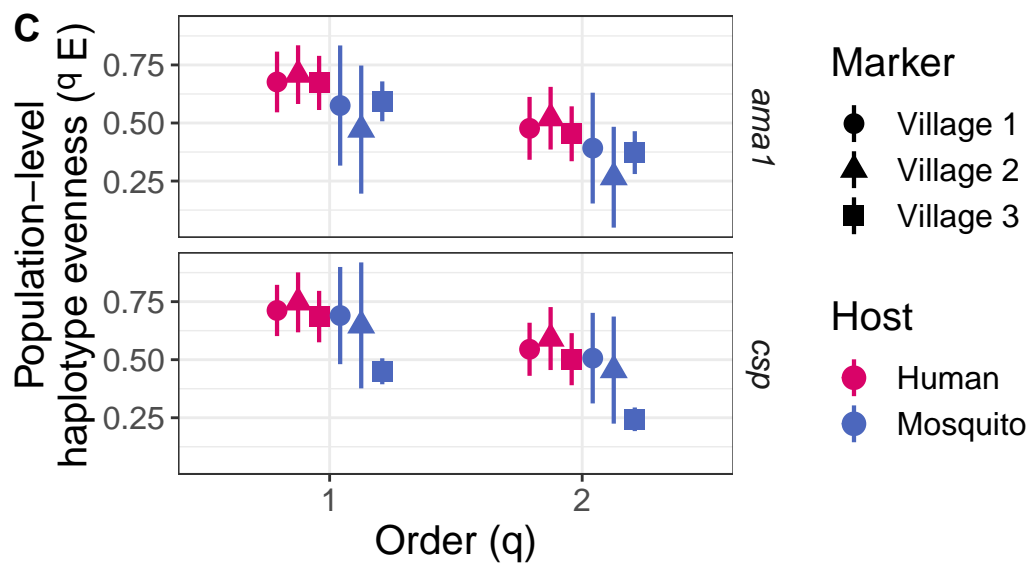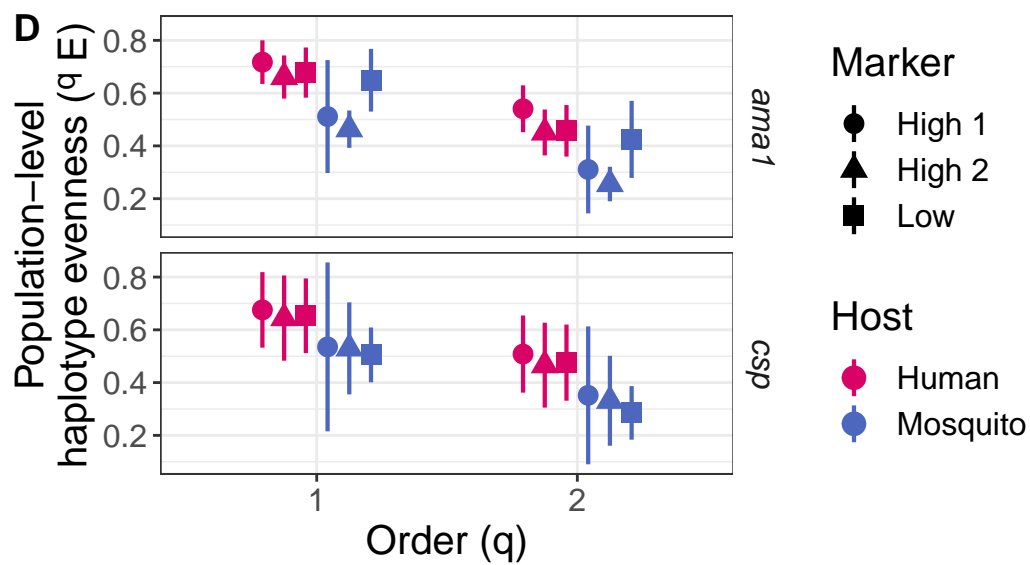

Supplement: FIG S7 [file mbio.02277-22-s0007.pdf]

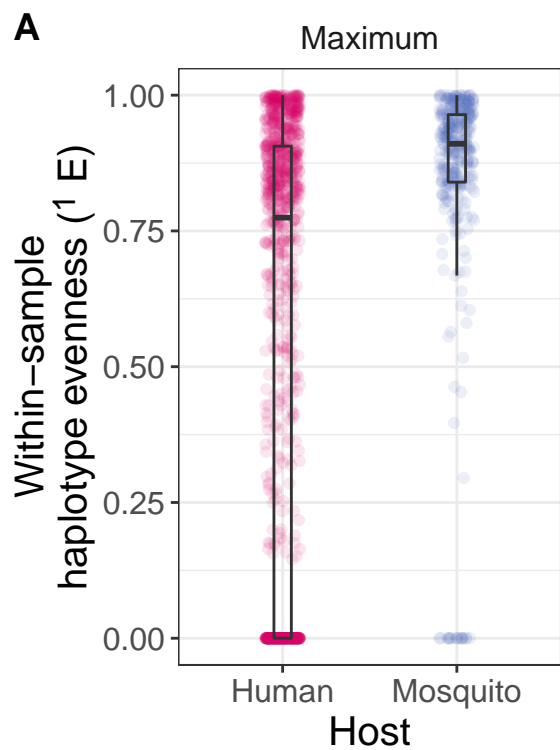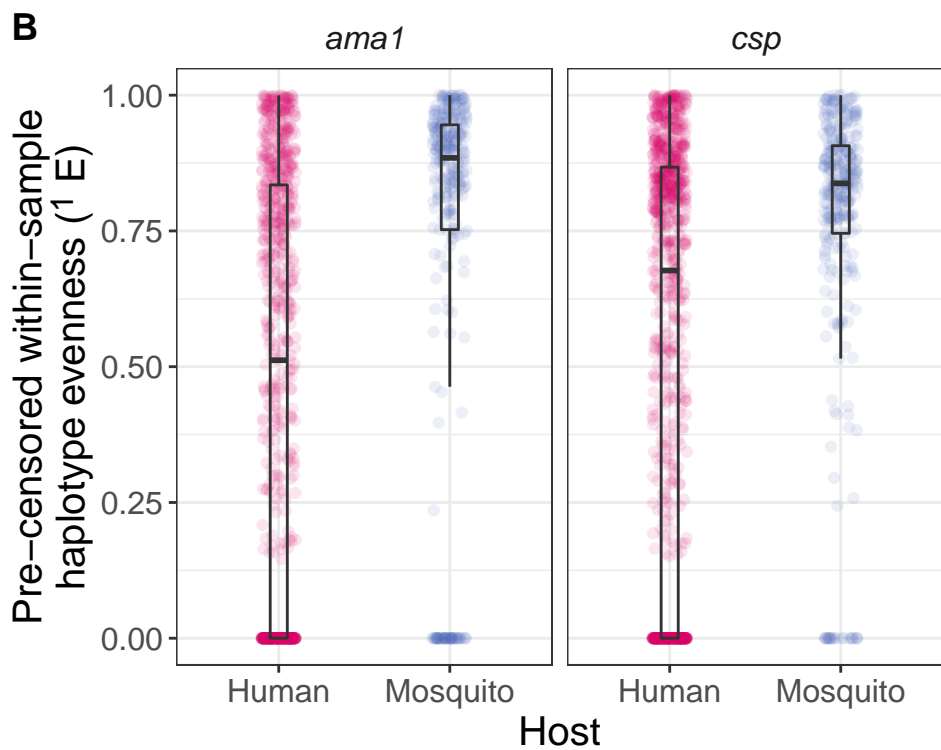

Supplement: FIG S8 [file mbio.02277-22-s0008.pdf]
