# Supplementary material for: Plasmodium falciparum Genetic Diversity in Coincident Human and Mosquito Hosts
Source: mBio. 2022 Sep 8;13(5):e02277-22. doi: 10.1128/mbio.02277-22 (PMC9600619; doi:10.1128/mbio.02277-22)
Supplement: FIG S4 [file mbio.02277-22-s0004.pdf]

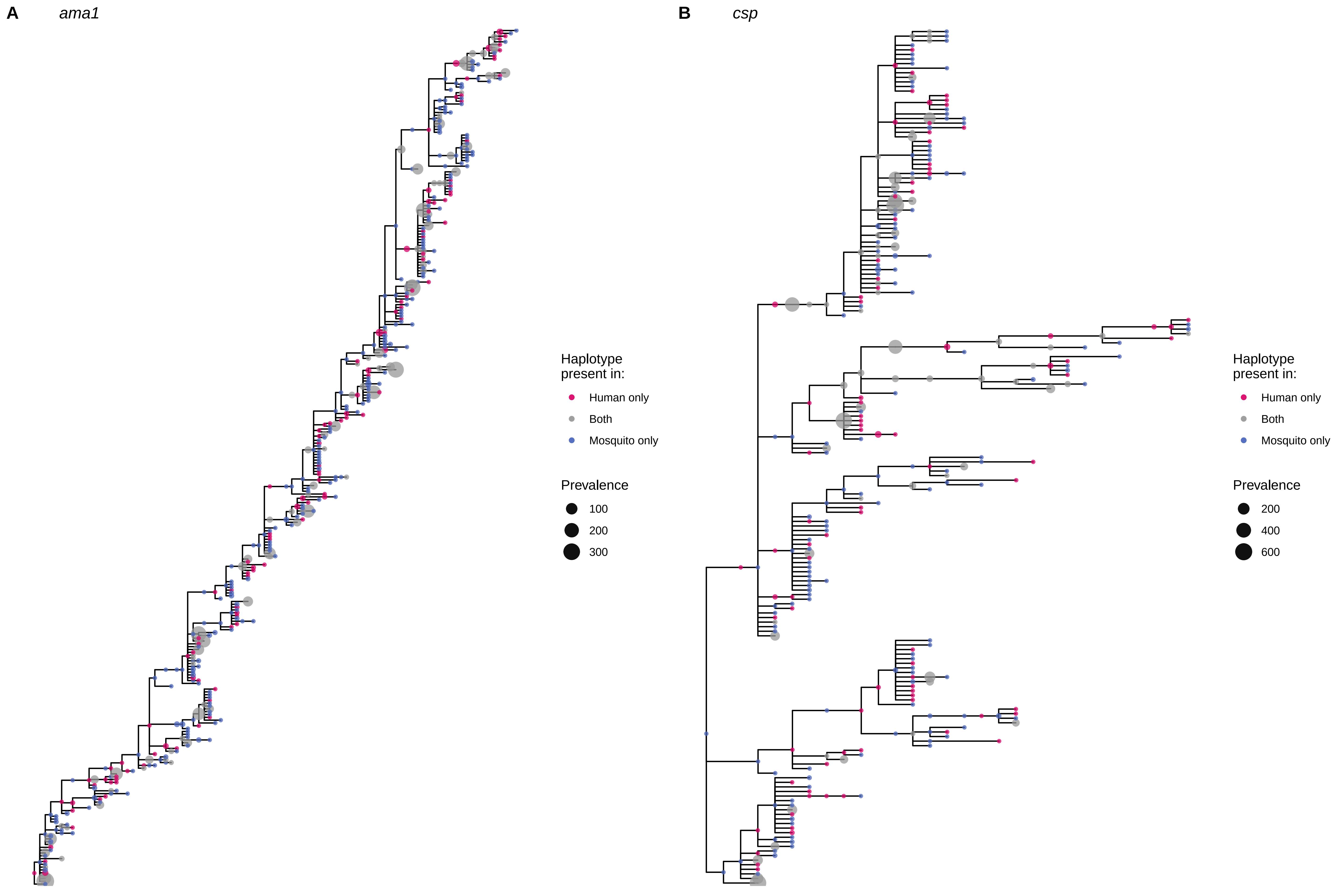
A
ama1
Haplotype present in:
Human only
Both
Mosquito only
Prevalence
100
200
300
B
csp
Haplotype present in:
Human only
Both
Mosquito only
Prevalence
200
400
600
