# Supplementary material for: Plasmodium falciparum Genetic Diversity in Coincident Human and Mosquito Hosts
Source: mBio. 2022 Sep 8;13(5):e02277-22. doi: 10.1128/mbio.02277-22 (PMC9600619; doi:10.1128/mbio.02277-22)
Supplement: FIG S6 [file mbio.02277-22-s0006.pdf]

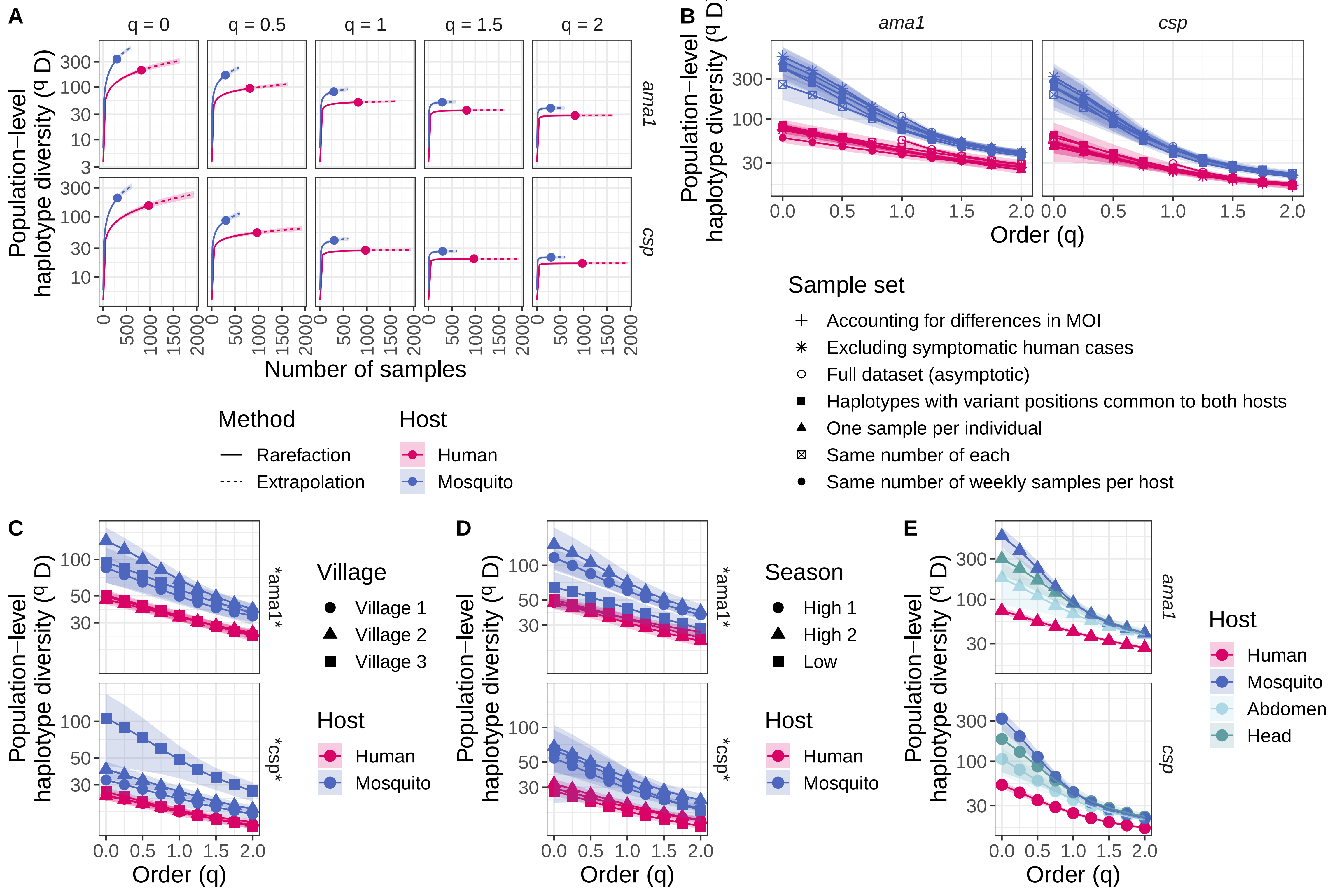
A
q = 0
q = 0.5
q = 1
q = 1.5
q = 2
ama1
csp
Population−level haplotype diversity (q D)
Number of samples
Method
Rarefaction
Extrapolation
Host
Human
Mosquito
B
ama1
csp
Order (q)
Sample set
Accounting for differences in MOI
Excluding symptomatic human cases
Full dataset (asymptotic)
Haplotypes with variant positions common to both hosts
One sample per individual
Same number of each
Same number of weekly samples per host
C
ama1*
csp*
Village
Village 1
Village 2
Village 3
D
Season
High 1
High 2
Low
E
Abdomen
Head
